# Identifying Gender-Preferred Communication Styles within Online Cancer Communities: A Retrospective, Longitudinal Analysis

**DOI:** 10.1371/journal.pone.0049169

**Published:** 2012-11-14

**Authors:** Kathleen T. Durant, Alexa T. McCray, Charles Safran

**Affiliations:** 1 Beth Israel Deaconess Medical Center, Boston, Massachusetts, United States of America; 2 Harvard Medical School, Boston, Massachusetts, United States of America; 3 Silverlink Communications, Boston, Massachusetts, United States of America; The University of Hong Kong, Hong Kong

## Abstract

**Background:**

The goal of this research is to determine if different gender-preferred social styles can be observed within the user interactions at an online cancer community. To achieve this goal, we identify and measure variables that pertain to each gender-specific social style.

**Methods and Findings:**

We perform social network and statistical analysis on the communication flow of 8,388 members at six different cancer forums over eight years. Kruskal-Wallis tests were conducted to measure the difference between the number of intimate (and highly intimate) dyads, relationship length, and number of communications. We determine that two patients are more likely to form an intimate bond on a gender-specific cancer forum (ovarian *P* = <0.0001, breast *P* = 0.0089, prostate *P* = 0.0021). Two female patients are more likely to form a highly intimate bond on a female-specific cancer forum (Ovarian *P*<0.0001, Breast *P*<0.01). Typically a male patient communicates with more members than a female patient (Ovarian forum *P* = 0.0406, Breast forum *P* = 0.0013). A relationship between two patients is longer on the gender-specific cancer forums than a connection between two members not identified as patients (ovarian forum *P* = 0.00406, breast forum *P* = 0.00013, prostate forum P = .0.0003).

**Conclusion:**

The high level of interconnectedness among the prostate patients supports the hypothesis that men prefer to socialize in large, interconnected, less-intimate groups. A female patient is more likely to form a highly intimate connection with another female patient; this finding is consistent with the hypothesis that woman prefer fewer, more intimate connections. The relationships of same-gender cancer patients last longer than other relationships; this finding demonstrates homophily within these online communities. Our findings regarding online communication preferences are in agreement with research findings from person-to-person communication preference studies. These findings should be considered when designing online communities as well as designing and evaluating psychosocial and educational interventions for cancer patients.

## Introduction

Early in life and continuing into adulthood, humans like other animals, segregate themselves by gender [1]–[7]. Within their segregated milieus, females prefer to interact with one or two other individuals at a single time in distinct and separate cliques [8]–[11]. Whereas males prefer to form one large interconnected group of many individuals; hence displaying a preference to socialize in large coalitions with dominance hierarchies. A smaller social sphere for females, allows females to allocate greater investment into fewer more intimate bonds [12]. These more intimate bonds that females form typically require many exchanges; more exchanges than male bonds [13]. Female to female relationships require more maintenance behavior than male to male relationships; maintenance behaviors such as more interaction, more openness and more supportiveness [14]. Given this required extra maintenance behavior, female relationships have been found to be more fragile than male bonds, requiring more of an investment of the two individuals [15]. A male to male bond has been shown to be more resilient and to last longer [16]–[18]. These different social styles have been shown to effect the preferred problem solving tasks; males have been found to be more efficient at collaborative problem solving tasks; while woman are more efficient at dyadic sharing tasks [12] and avoidance of conflict [15].

Given these different male and female preferred social styles, we investigate if these different social styles can be identified within an online cancer community. We quantify gender-specific social styles by measuring the number of people a typical member communicates with and the number of shared messages between two members. Our findings show online communication styles of identified male patients typically follow the preferred communication styles associated with males and online communication styles of identified female patients typically follow the preferred communication style of females.

Research has shown that language (the chosen words) as well as discourse constructs used by women is different from language and discourse constructs used by men [19]–[20] and these differences can be found in face-to-face communication as well as computer-mediated communication. Computer-mediated communication has been analyzed using methodological techniques such as conversation analysis, critical discourse analysis, and language variation. It has been applied to electronic mail lists, Usenet newsgroups, chat room dialogs, and more recently online video sessions [21]–[25]. Other computer-mediated communication studies have focused on health communities [26]–[28]. These previous studies analyzed the communication content of a mixed-gender online health community; we consider the communication patterns between male-to-male and female-to-female online cancer forum members. Our communication pattern results dovetail with the prior communication content analysis studies; gender-specific communications patterns found within online communication patterns are similar to face-to-face communication patterns.

Identifying a female-specific and a male-specific communication style practiced by cancer patients within an online cancer forum allows the medical community to understand each gender’s preferred method for seeking and discovering information online as well as their method for seeking social support for coping with cancer. Previous research has analyzed social support for cancer patients both online and in person [29]–[43], our research considers the effect gender-preferred communication and social styles have on the social support cancer patients practice and provide within an online cancer forum. We believe monitoring patients’ communication interactions can provide insights into the different psychosocial needs of male and female cancer patients. These insights can be used to refine online social communities as well as gender-specific psychosocial interventions for cancer patients. For example, research on psychotherapy demonstrates that females prefer one-on-one counseling, whereas males prefer group counseling for topics such as substance abuse (Alcoholic Anonymous), post-traumatic stress disorder and sexual abuse [43]–[50]. This research shows gender-specific social preferences can also be found in online communities and should be considered when designing and evaluating Health 2.0 interventions.

We view communication interactions within an online medical forum as a patient-chosen psychosocial intervention. Patients are seeking medical advice, social support, and survival tactics from people online who have experience with the same disease. It is an activity that patients as well as caregivers are practicing to help cope with cancer. This practice has not necessarily been recommended by a health care professional, yet 18% of the US Internet users have turned to the Internet to find similar patients with the same medical condition [51]. Clinical research has reported benefits from discussing health issues with other people. For example, one study has shown that cancer patients who join in-person discussion groups experience a significantly improved quality of life, a significantly reduced pain level [33] as well as a decrease in the three most significant stressors for cancer patients: unwanted aloneness, loss of hope and loss of control [34]. One study reports a decrease in depression and reaction to pain for online support group members [31], [35].

Online cancer forums provide an opportunity to become part of a community where the common factor among the members is the battle against cancer. It also provides a method for cancer patients to offer peer support. Peer support allows people with similar experiences to offer each other practical advice and suggestions for strategies that professionals may not offer [52]. Online forums provide a benefit to patients seeking information (thread creators) through the information they receive. They also provide a benefit to patients providing information (thread responders) since these patients receive a sense of accomplishment by providing coping methods and knowledge on cancer that is beneficial to other cancer patients [52]. Peer social networks have been shown to improve the quality of life of participants [53]–[55].

## Materials and Methods

### Ethics Statement

Ethical approval: This work was approved by the Beth Israel Deaconess Medical Center Institutional.

Review Board. Since the research involved publicly available comments from the Internet, the board decided consent from the members providing the comments was not needed.

### Participants

Www.cancercompass.com is an active cancer forum for individuals interested in discussing issues associated with 33 different types of cancer as well as forums on nutrition, care giving, treatment and prevention of cancer. Cancercompass.com was created in 2001 by the Cancer Treatment Centers of America™. We examine the communication patterns between 8,388 members on six different cancer-specific forums consisting of 27,450 unique communication messages. We harvest the posts, threads and users’ data for the six cancer forums from cancercompass.com using html parsers. The collection was created on May 17, 2010; the time span of the thread corpus is from September 1, 2001 to April 30, 2010. We discarded threads created between April 30, 2010 and May 17, 2010 but allowed response posts to previously created threads to be included within the study. This allowed each forum at least 17 days to respond to an existing thread.

Table 1 describes the metadata associated with six different cancer forums found on the website: melanoma, renal cell carcinoma, breast cancer, ovarian cancer, testicular cancer and prostate cancer. The table describes the number of users and the user types found at each forum; the number of threads authored and the number of posts written by each of the different user types. We describe the collected data below.

**Table 1:** User type, thread and post count for the participating forums.

User Type chosen by Members at Registration									
User Type	Patient	Survivor	Care giver	Doctor	Nurse	Student	Researcher	Unknown	Total
**Member Groups**									
	**Patient/Survivors**		**Non-patients**						
**Forums**									
Melanoma	351	54	155	8	1	0	29	367	965
Renal-cell	327	21	232	6	2	0	7	308	903
Prostate	609	81	231	31	0	0	47	378	1377
Testicular	21	8	15	3	0	0	11	39	97
Ovarian	554	129	99	15	0	1	31	518	1347
Breast	1453	587	161	36	1	1	67	976	3282
**Total**									8388
**Threads**									
Melanoma	205	22	81	3	1	0	17	273	602
Renal-cell	216	8	195	2	1	0	3	248	673
Prostate	565	38	194	4	0	0	39	343	1183
Testicular	13	2	10	00	0	0	11	31	67
Ovarian	350	42	62	3	0	1	27	376	861
Breast	956	309	69	8	0	0	37	620	1999
**Total**									5385
**Posts**									
Melanoma	1003	117	452	122	5	0	39	986	2724
Renal-cell	1323	138	1145	20	6	0	12	923	3567
Prostate	3346	790	657	411	0	0	121	1146	6471
Testicular	37	10	21	6	0	0	13	58	145
Ovarian	2919	386	333	42	0	1	70	1173	4924
Breast	4841	2158	301	241	2	1	113	1962	9619
**Total**									27450

A member is a user or a person within the discussion forum; a user may be one of several different user types: *caregiver*, *patient*, *survivor*, *doctor*, *nurse*, *student*, *researcher* and *unknown*. Members self-assign a user type when they register at the cancercompass website. A user type typically describes the relationship the user has with cancer. Since user type is the only data collected that describes the member, we use this data as well as the cancer type to deduce the gender of ovarian cancer and breast cancer patients as females and testicular and prostate patients as males. Within our statistical analysis, we group members who register as caregivers, doctors, students, researchers, nurses and unknown as members of the *non-patient* group and members who register as survivors and patients as the *patient/survivor group*. We use these categorical variables as factors within our statistical analysis.

A thread is created when one member poses a discussion topic and other members post text relating to the topic. A member who poses a discussion to the forum is the creator or the author of a thread. A thread is a discussion that is open and may be joined by any existing member. Both the number of threads and the number of posts are listed in Table 1.

There is a large variation between the sizes of the six forums in all three measures (number of users, threads, posts). This is expected since the prevalence of these cancers varies. Another interesting variation among the forums is the percentages of members who have been diagnosed with cancer that consider themselves a survivor rather than as a patient. The percentages of survivors of the members who have been diagnosed with cancer are: melanoma forum 13.3%, renal cell cancer forum 6.0%, prostate cancer forum 11.7%, ovarian cancer forum 18.9%, and breast cancer forum 28.8%. The patients on the female-specific cancer forums are more likely to identify with the label *survivor* when compared to the other forums. Given the limited communication on the testicular forum (145 posts in eight years), we eliminate it from the statistical analysis.

### Factors Affecting Analysis

Since we are investigating gender specific behaviors, in Table 2 we present Surveillance Epidemiology and End Results (SEER) age-adjusted incidence rate (time period for diagnosis 1975–2007) for each studied cancer stratified by gender; the data was tabulated by the U.S. National Cancer Institute [56]–[58]. Table 2 shows that males are more than twice as likely to be diagnosed with renal cell cancer as females, whereas the melanoma incidence is more balanced between the two genders. We hypothesize that the difference in gender incidence between these two cancers will affect the communication patterns found at these gender-neutral forums. Given the high percentage of renal cell cancer patients that are male, we expect the communication style of a renal cell cancer patient to more likely follow the behavior of a male cancer patient than a female cancer patient. Given the low number of males diagnosed with breast cancer, we treat the breast cancer forum as a female-specific cancer forum.

**Table 2:** Cancer incidence rate per 100,000.

	Breast	Ovarian	Prostate	Testicular	Melanoma	Renalcell
MaleIncidence	1.08	0	154.25	5.04	18.83	15.21
FemaleIncidence	124.68	14.75	0	0	13.12	7.46

### Network Creation

We represent each cancer forum as a social network [59]–[60] where the nodes represent the members of the forum and the arcs represent the directed communication between two members. A node is added to the network when that member writes his/her first post. A connection or arc between two nodes represents a directed communication channel between two members and constitutes a bond between the members. The relative thickness of an arc represents the number of directed communications between the two members and is an indicator of the bond’s intimacy. The software network tool Pajek version 2.04 [59] is used to represent the networks; the Kamada-Kawai algorithm [61] is used to visualize the networks.

### Methods

We measure the total number of messages a member composes; this value corresponds to the total contribution this person has made to the forum. It is represented in the network by the total number of output edges as well as the edges’ thickness emanating from a node. We also measure the number of members each member has corresponded with (breadth variable). This value is represented in the network by the number of edges connected to a node. These two communication metrics allow us to distinguish members who prefer to communicate with a small group of people from members who prefer to communicate with a relatively larger group of people.

As in previous studies, we represent the level of intimacy between two forum members with the total count of communication interactions between the two members [62]–[63]. We convert the count of communication interactions to an ordinal variable representing three different relationship levels: *acquaintance*, a *slightly intimate* relationship and a *highly intimate* relationship. The relationship level is a proxy for the level of support the two members are providing to one another; it differentiates the intimate relationships from the casual relationships.

We define the different relationship levels as the following. Two members communicating fewer than the average plus the standard deviation are considered acquaintances. Members communicating more than this threshold are considered intimate. We further separate the intimate connections into slightly intimate and highly intimate connections. An intimate member, communicating more than the average plus two times the standard deviation, is considered highly intimate. A highly intimate relationship identifies two members who prefer to communicate with one another. We measure the likelihood that two patients will have an intimate or highly intimate relationship to determine if patients prefer to communicate online with other patients rather than other online users.

### Statistical Methods

Since the variables we measure are not normally distributed, we use nonparametric tests for statistically significant testing. Also, since we are performing multiple pairwise comparisons, we employ the Bonferroni correction method when performing multiple comparisons. We use the Kruskal-Wallis test for analysis; since the Kruskal-Wallis test returns differences on the ranks of the means, only the direction (positive, negative) of the difference can be used in the interpretation of the results.

## Results

### Statistical Analysis

Table 3 presents a comparison of the communication metrics of the patient/survivor group (members registering as either a patient or a survivor of cancer) within the five forums. Each row in Table 3 performs a pair-wise comparison of the number of patient/survivor connections and the number of messages communicated by patient/survivors on each of the five forums. These two metrics quantify the number of people a patient/survivor communicates with (breadth of a member’s connections) and the total number of messages created by a patient/survivor (contribution to the forum). When comparing the number of connections for patient/survivors using forum as a factor, (column 2, *P* column), Table 3 shows patient/survivors in the ovarian and the breast forum (identified female patient/survivors) statistically behave the same (row 5). They also behave similarly, in terms of the number of people communicated with and the total number of created messages, as the melanoma patient/survivors (row 7, row 9).

**Table 3:** Communication metrics comparison on the six forums.

		Number of patient connections		Number of patient messages	
Row	Forum comparison	Difference	*P*	Difference	*P*
1.	Prostate vs. Ovarian	175.70	.0406	172.03	0.0593
2.	Prostate vs. Breast	191.41	.0013	195.47	0.0013
3.	Prostate vs. Renal cell	36.48	1.00	7.23	1.000
4.	Prostate vs. Melanoma	276.64	.0010	292.75	0.0005
5.	Ovarian vs. Breast	15.70	1.00	23.44	1.0000
6.	Ovarian vs. Renal cell	−139.23	.6200	−164.79	0.3071
7.	Ovarian vs. Melanoma	100.93	1.00	120.72	.9641
8.	Breast vs. Renal cell	−154.92	.1837	−188.23	0.0507
9.	Breast vs. Melanoma	85.23	1.00	97.28	1.0000
10.	Renal cell vs. Melanoma	240.16	.0373	285.51	0.0075

Table 3 shows that patient/survivors on the prostate forum, in general, choose to communicate with more members than patient/survivors of the breast, ovarian and melanoma forums (row 1, row 2, and row 4 respectively). The number of connections for the renal cell cancer patient/survivors does not statistically differ from the prostate and the ovarian forums (row 3, row 6) but does differ from the melanoma patient/survivor connections and the breast cancer patient/survivor connections (row 8, row 10). Prostate patient/survivors send more messages than breast cancer patient/survivors and melanoma patient/survivors (row 2 and row 4 column 4). Male prostate patient/survivors within the prostate forum send more messages and connect with more people than female patient/survivors do on the breast cancer forum. However, female ovarian patient/survivors send statistically the same number of messages as prostate patient/survivors but these ovarian patient/survivor messages are sent to fewer people.

Table 4 compares the relationship variables of the patient/survivor population to the general population for each of the five forums. It is not comparing measures across forums as the analysis in Tables 3 did. It measures the relationship duration (measured in days), for the intimate and the highly intimate communications between patient/survivor dyads (both members registered as a patient or a survivor) versus other dyads where both members are not patient/survivors (at least one of the members registered as a caregiver, doctor, nurse, student, researcher or other).

**Table 4:** Relationship metric comparisons at the six forums.

	Relationship durationPatient vs. nonspecific		Intimate connectionsPatient vs. nonspecific		Highly Intimate connectionsPatient vs. nonspecific	
	Diff	*P* value	Diff	*P* value	Diff	*P* value
Prostate	83.88	0.0006	48.51	0.0021	6.648	.1985
Ovarian	67.28	0.0008	54.43	<0.0001	9.5	<.0001
Breast	101.05	0.0003	37.66	0.0089	20.40	<.01
Melanoma	−65.15	0.0022	−20.01	.0206	NA	
Renal cell	−16.29	0.4869	1.2466	.9224		

Column 1, Table 4 shows that a relationship between two patient/survivors is more likely to be longer (in days) on the prostate, ovarian, and breast forums (gender-specific cancer forums) than a connection between two members where both are not identified as patient/survivors. Within the renal cell cancer forum there is no significant difference between the relationship duration of two patient/survivors when compared to the duration of a typical connection between two non-patient/survivor members. Melanoma patient/survivors are more likely to have shorter connections (in days) than two members not identified as patient/survivors. This finding shows that same-gendered patient/survivors, once connected, will stay connected longer to each other than to a member who is a non-patient member.

Column 2, Table 4 shows that an intimate connection on the prostate, ovarian, and breast cancer forums is more likely to exist between two patient/survivors than between two members not identified as patient/survivors (*P* = .0021, *P*<.0001, *P* = .0089 respectively). Within the renal cell forum there is no statistical difference between the types of members forming an intimate connection (*P* = .9224). Within the melanoma forum, two patient/survivors are less likely to form an intimate connection than two members who both are not patient/survivors (negative result, *P* = .0206). This finding supports the belief that same gender patient/survivors suffering from the same cancer, will prefer to communicate with another patient/survivor with the same gender (gender homophilic effect).

Since column 3, Table 4 shows that an intimate connection is more likely to exist between two patient/survivor members on a gender-specific forum, we next determine if highly intimate connections are also more likely to exist between two patient/survivors on these gender-specific cancer forums. Column 3, Table 4 shows that an intimate connection is more likely to exist between two patient/survivors on a female-specific cancer forum (breast *P*<.01, ovarian *P*<.0001); however a male patient/survivor on the prostate forum is not more likely to form a highly intimate connection with another male patient/survivor (*P* = .1985). This finding shows that female patient/survivors on female-specific cancer forums are more likely to form a highly intimate connection with one another; however male patient/survivors on male-specific forums are not more likely to form a highly intimate connection with another male patient/survivor. This finding supports the belief that female to female relationships require more maintenance behavior than male to male relationships; maintenance behaviors such as more interaction, more openness and more supportiveness [14].

### Social Network Analysis

**Figure 1:**
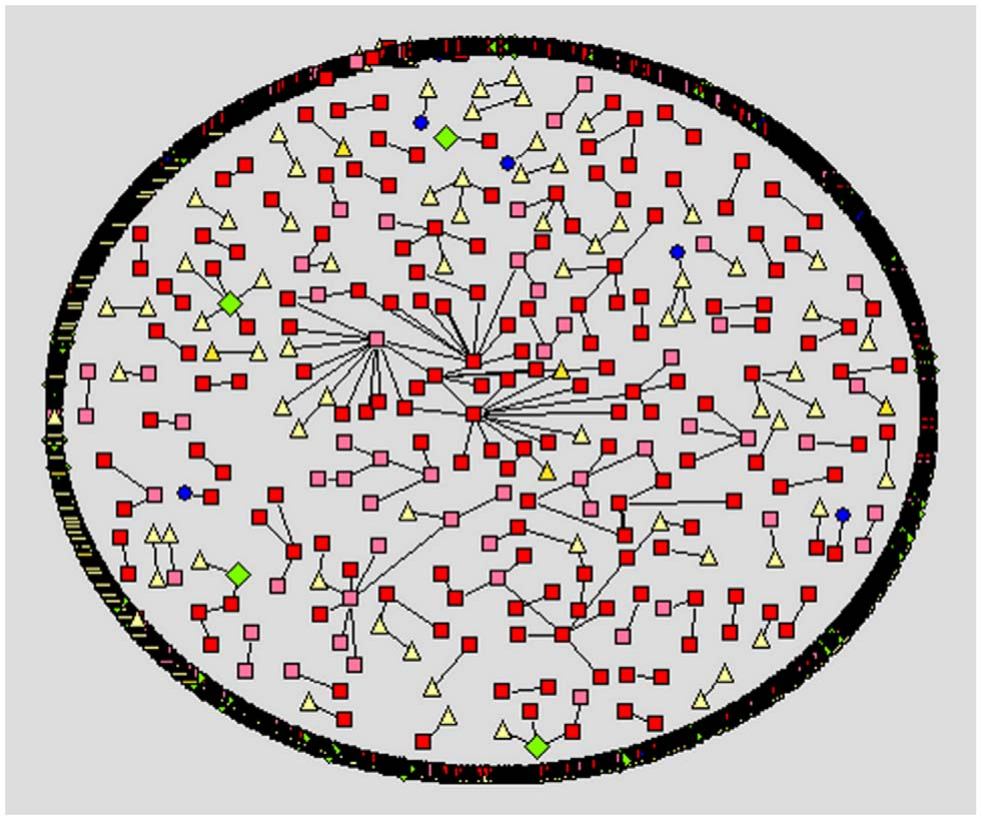
Breast cancer forum, intimate dyads.

**Figure 2:**
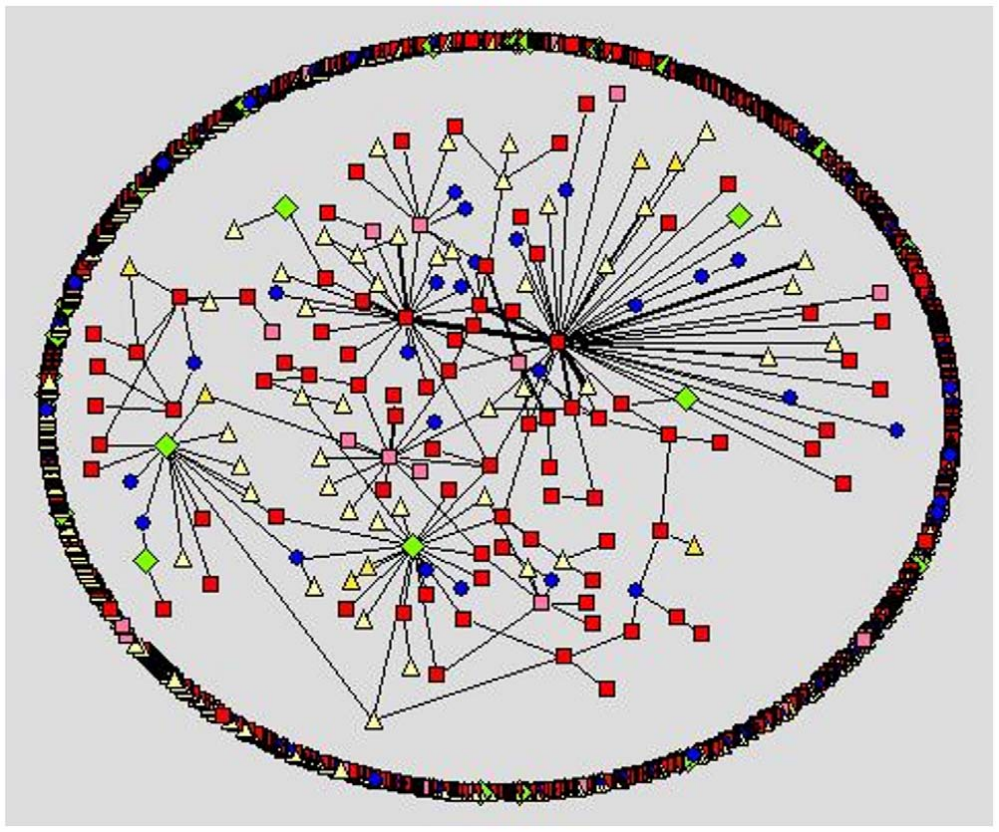
Prostate cancer forum, intimate dyads.

**Figure 3:**
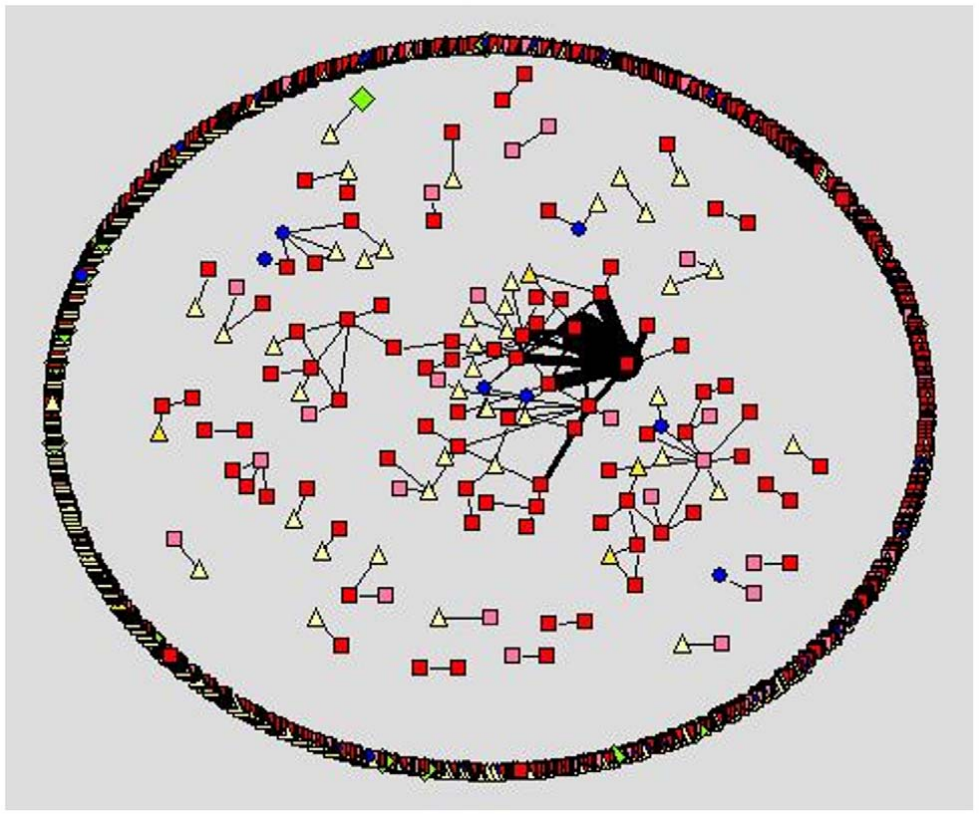
Ovarian cancer forum, intimate dyads.

**Figure 4:**
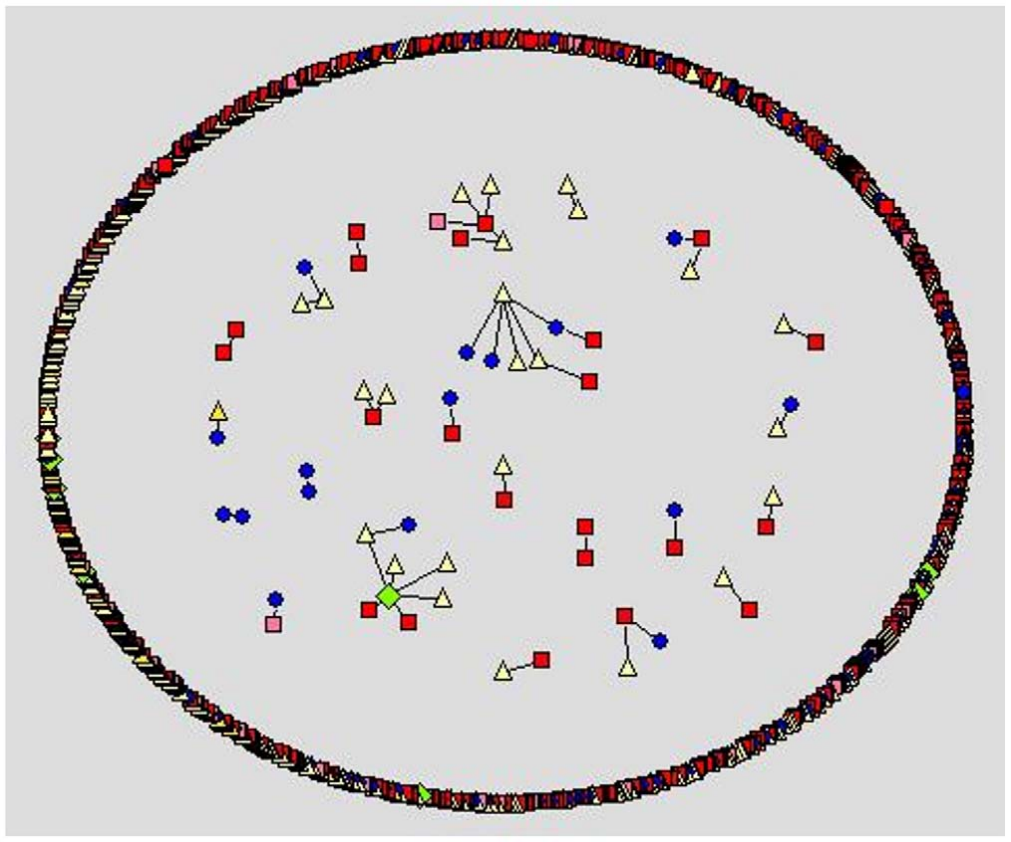
Melanoma forum, intimate dyads.

**Figure 5:**
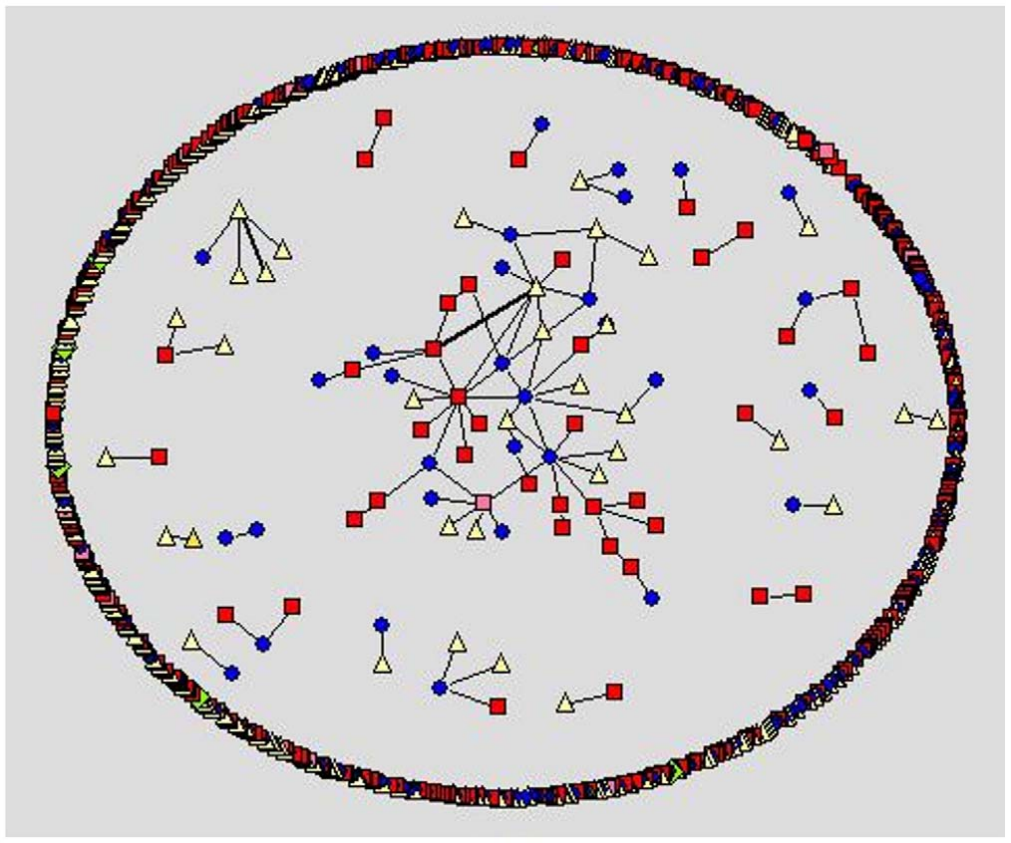
Renal cell cancer forum, intimate dyads.

We apply social network analysis [59]–[60] to visualize the different social styles within the forums. Within the visualization, we use different node color and shape for the different user types a person may register as at the cancercompass website. However, we use shades of red to represent the patient/survivor group. In Figures 1–5 the representation is the following: red square nodes are patients, pink square nodes are survivors, blue circle nodes are caregivers, light yellow triangle nodes are unknown, and light green triangle nodes are doctors. The relative thickness of an arc represents the number of communication messages between two members; a thicker arc represents more communication. We limit the visualization to intimate relationships, since we are interested in the small sub-network that may feel a social connection to one another. Interestingly, none of the members registered as a nurse, researcher or a student are part of an intimate dyad.

The breast cancer forum (Figure 1) is the largest forum and contains the highest number of intimate connections. Its intimate connections are primarily between two patient/survivors. Within the gender-specific cancer forums (Figures 1–3), a patient/survivor member are more likely to have an intimate connection with another patient/survivor member. This fact is visually displayed by the prominence of red and pink nodes within the gender-specific cancer forums. This finding is not found in the gender-neutral cancer forums. In the gender-neutral cancer forums (Figure 4–5) caregivers are playing an important role in the formation of intimate connections. Unfortunately, we do not have access to the caregivers’ genders so we are unable to determine if there is a specific gender associated with the majority of these caregivers. This fact is visually displayed by the balance of red and blue nodes within Figure 4 and Figure 5.

Within the ovarian cancer forum (Figure 3) we observe many, relatively thicker edges between patients/survivors, demonstrating the highly intimate relationships between these two female patients/survivors. Within the prostate cancer forum (Figure 2) there are few disconnected sub-networks. All nodes are directly or indirectly connected to one another. This displays the high level of interconnectedness among male patients in the prostate forum. Male patients prefer to discuss topics with many different people. This is very different from the breast cancer forum where there are many sub-networks of two to four people only connected to each other. There are a few sub-networks within the center of the network where some nodes are connected to more than four people but typically the network consists of many disconnected sub-networks (Figure 1).

## Discussion

The communicative behavior of a male cancer patient/survivor on a male-specific cancer forum is typically different from the communicative behavior of a female patient/survivor on a female-specific cancer forum. Patient/survivors within the prostate forum, in general, connect with more members than female patient/survivors do on the ovarian and breast cancer forums. This finding is visually displayed in Figure 2 and statistically shown in Table 3. This supports the belief that men prefer to socialize in large groups. Identified male patient/survivors communicating in online cancer forums are displaying aspects of the preferred face-to-face communication style of males. This social preference is practiced in group therapy, which has been shown to work well with men [45]–[50].

The communicative behavior of a female patient/survivor within a female-specific cancer forum is different from the behavior of a male patient/survivor on a male-specific cancer forum. A female patient/survivor on a female-specific cancer forum (ovarian cancer or breast cancer) is more likely to form a highly intimate connection with another female patient/survivor. This finding is visually displayed in Figure 1 and Figure 3 and statistically shown in Table 4. A highly intimate connection means more communication between two specific female patient/survivors. Female patient/survivors are choosing to communicate more messages to a select group of other female patient/survivors. This supports the belief that females prefer to communicate heavily (number of messages) with fewer people (number of nodes) [12]–[14]. Identified female patient/survivors communicating in online cancer forums are displaying aspects of the preferred communication styles of females. This social preference should be modeled when defining Health 2.0 interventions for females; for example providing multimodal, omnipresent forms of communication (such as SMS, or Instant Messaging). Also providing private areas and/or times when two female patient/survivors may meet in person and discuss their illness alone should also be considered.

Women diagnosed with breast cancer or ovarian cancer are significantly more likely to register as a survivor at the cancercompass website than a cancer patient at the prostate cancer, melanoma or renal cell cancer forum. This finding is difficult to interpret since there are many different definitions of a cancer survivor [64]. One definition defines a person as a cancer survivor from diagnosis until the end of his/her life [64], [65]; whereas another definition limits survivors to any person diagnosed with cancer in the past but has gone beyond his/her initial treatment and have no evidence of the disease [64]. If women are using the former definition then they are categorizing themselves as fighting the disease. However, if they are using the latter definition, then more women who have lived beyond cancer are choosing to join the online community to support women currently battling cancer. Given these varying definitions for the term survivor, we are not able to determine the significance of the percentage difference; however we can state that female cancer patients are accepting the identity of a cancer survivor.

The length of the relationship between two patient/survivors in the gender-specific forums is longer (in days) than the relationships between two members not identified as patient/survivors; however this is not found to be true for patient/survivors within a gender-neutral cancer forum. Patient/survivors with the same gender, suffering from the same cancer have longer relationships (measured in days) than relationships between two non-patients found on the same forum. Same-gender patient/survivors suffering from the same cancer once connected, communicate with each other for a longer period of time. This finding supports the belief that patient/survivors join online cancer forums to communicate with people who are or have experienced what they are experiencing; and are similar in ilk to them; the homophilic or birds-of-a-feather phenomenon [57]. However, since patient/survivors do not exhibit this behavior on gender-neutral forums, we believe gender plays a crucial role in online relationship creation and longevity. It may also play a role in online peer support among cancer patients.

Even though we have categorized the melanoma and renal cell forums as gender-neutral forums, they are statistically different from each other for communication measures. Our analysis shows that the renal cell forum measures are more similar to the prostate response measures than to the measures of the melanoma forum. A typical renal cell cancer patient/survivor communicates with the same number of forum members as the prostate cancer forum member. We believe this is due to the likelihood of more male forum members on the renal cell forum given the high incidence rate of renal cell carcinoma in males compared to females (Table 2). However, since we do not have gender specifications for these members we cannot verify this hypothesis.

For the relationship measures, the gender-neutral forum measures are different from the gender-specific forum measures. Male patient/survivors on a male-specific cancer forum and female patient/survivors on the female-specific cancer forums are more likely to have an intimate bond with another patient/survivor. This is not true for a patient/survivor on a gender-neutral cancer forum. A renal cell cancer patient/survivor is just as likely as any other forum member to form an intimate bond with another member. A melanoma patient/survivor’s relationship measures are lower than a typical member’s relationship measures, meaning two melanoma patient/survivors are less likely to be intimate on the melanoma forum. This finding may be because the different gender-preferred communication styles or different language styles [21]–[28] are preventing patient/survivors with different genders from bonding. It may also be due to the strong influence of the caregiver members as demonstrated in Figure 5. Melanoma patients may have a stronger support network than a typical cancer patient; such as an at-home caregiver willing to engage online with other people discussing their loved one’s melanoma.

As found in this research as well as other studies, males and females typically have different communications preferences; these preferences can influence the results of a Health 2.0 communication interventions research study. For example, there are many studies evaluating the use of SMS technology for adherence to treatment; adherence topics such as weight management [66]–[67], diabetes treatment [68]–[71], HIV treatment [72]–[73], breast cancer screenings [74], and sunscreen application [75]–[76]. It is important that these studies stratify the results by gender, to recognize the actual benefits of the study. Also it may be difficult to choose one optimal number of outreaches for both genders, since the two genders typically prefer diverse communication styles. An optimal number of outreaches for a female may be too many for a typical male.

### Limitations of the Study

Unfortunately the descriptive information we have associated with each member is limited to a user type. In particular, we do not have access to the gender information for each member. We can only deduce the gender for a subset of the patients given the cancer type diagnosis, for example members registering as a patient or a survivor on a male cancer forum is a man, and a member registering as a patient or a survivor on a female cancer forum is a woman. If we had access to each member’s gender, we could differentiate between the varying homophilic tendencies of same gender and same cancer diagnosis among patients. We also do not have access to a participant’s age, state of residence, educational level, socio-economic, medical history, technical capacities or other describing features that may influence a person’s participation in an online health community. These factors may affect the outcome measures.

Our study assumes the number of communication interactions represent the level of intimacy between two members. Even though this representation has been used in prior studies, it has not been validated as a proxy for intimacy. For example, the content of a message such as the topics discussed could provide a more accurate representation of intimacy.

Our study is limited to one online website (www.cancercompass.com) and six online communities within this web site (breast cancer forum, ovarian cancer forum, prostate cancer forum, testicular cancer forum, renal cell cancer forum and melanoma forum); the website may not be representative of other websites that host online cancer communities. The male-specific online communities within this study may not be representative of typical interactions found at other male-specific online health communities. The female-specific online communities within this study may not be representative of typical interactions found at other female-specific online health communities.

For statistical analysis, we grouped patients and survivors into one group since we wanted to investigate communication patterns between people who have had a cancer diagnosis vs. people who support people with a cancer diagnosis. However people considering themselves a survivor may behave differently than people who consider themselves a patient.

### Conclusion

We have identified male-preferred social styles being conducted by male patient/survivors in a male-specific cancer forum and female-preferred social styles being conducted by female patient/survivors. Males prefer a high level of interconnectedness; this supports the belief that men prefer to socialize in large, interconnected groups. Females are more likely to form a highly intimate connection with another female and the sub-network consisting of females within the female-specific forums provide a lower level of interconnectedness than the prostate forum. These findings support the belief that women prefer fewer, more intimate connections within their social group. Identifying and understanding patients’ communicative profiles will help quantify the informational and relational support desired by female and male cancer patients. These findings can be useful when designing educational and psychosocial interventions for cancer patients. Lastly, monitoring the behavior of male and female cancer patients within online cancer forums can provide insight into the different psychosocial support needed by male cancer patients and female cancer patients.
